# 1-[5-(Anthracen-9-yl)-3-(4-nitro­phen­yl)-4,5-dihydro-1*H*-pyrazol-1-yl]ethan-1-one

**DOI:** 10.1107/S1600536810053365

**Published:** 2011-01-15

**Authors:** Bao-Li Dong, Ming-Liang Wang, Yong-Hua Li

**Affiliations:** a School of Chemistry and Chemical Engineering, Southeast University, Nanjing 211189, People’s Republic of China

## Abstract

In the title compound, C_25_H_19_N_3_O_3_, steric repulsion between the methine H atom and one of the anthryl H atoms seems to be concomitant with the considerable distortion of the anthryl fragment from planarity. The side rings of the anthryl subtend an angle of 9.57 (8)°, which is an extreme value among the known reliably determined structures. This angle correlates with the length of the bond by which the anthryl is attached to the rest of the mol­ecule. In the anthryl fragment, the maximum deviation of one of the C atoms from the mean plane is 0.126 (3) Å and regards the carrier C atom involved in the repulsion between the anthryl and the methine H atoms. The inter­planar angle between the pyrazoline ring and the anthryl fragment is 88.36 (5)° and that between the pyrazoline and 4-nitro­phenyl rings is 8.80 (15)°. Weak inter­molecular C—H⋯N, C—H⋯π and π–π inter­actions [centroid–centroid distances of 3.7659 (17), 3.9477 (15) and 3.8972 (15) Å] are pesent in the structure.

## Related literature

For the related structure 1′,2′,3′,4′-tetra­hydro-1,3-diphenyl-4-*p*-tolyl­spiro­[2-pyrazoline-5,2′-naph­thalen]-1′-one, see: Krishna *et al.* (1999 ▶). For examples of the synthetic utility applied in the case of the title compound, see: Akama *et al.* (1996 ▶); Fahrni *et al.* (2003 ▶); Wei *et al.* (2007 ▶). For a description of the Cambridge Structural Database, see: Allen (2002 ▶).
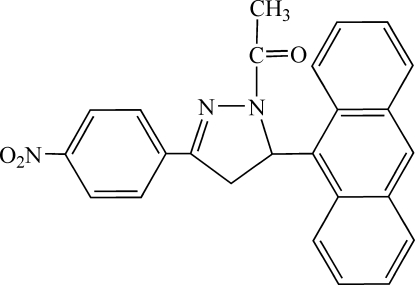


## Experimental

### 

#### Crystal data

C_25_H_19_N_3_O_3_
*M* *_r_* = 409.43
Orthorhombic, 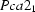

*a* = 22.888 (5) Å
*b* = 9.4031 (19) Å
*c* = 8.9688 (18) Å
*V* = 1930.3 (7) Å^3^
*Z* = 4
Mo *K*α radiation
μ = 0.09 mm^−1^
*T* = 293 K
0.30 × 0.20 × 0.20 mm

#### Data collection

Rigaku SCXmini diffractometer
Absorption correction: multi-scan (*CrystalClear*; Rigaku, 2005 ▶) *T*_min_ = 0.962, *T*_max_ = 0.982
19272 measured reflections
2352 independent reflections
2131 reflections with *I* > 2σ(*I*)
*R*_int_ = 0.062

#### Refinement

*R*[*F* ^2^ > 2σ(*F* ^2^)] = 0.039
*wR*(*F* ^2^) = 0.097
*S* = 1.07
2352 reflections
288 parameters
69 restraints
H atoms treated by a mixture of independent and constrained refinement
Δρ_max_ = 0.17 e Å^−3^
Δρ_min_ = −0.18 e Å^−3^

### 

Data collection: *CrystalClear* (Rigaku, 2005 ▶); cell refinement: *CrystalClear*; data reduction: *CrystalClear*; program(s) used to solve structure: *SHELXS97* (Sheldrick, 2008 ▶); program(s) used to refine structure: *SHELXL97* (Sheldrick, 2008 ▶); molecular graphics: *SHELXTL/PC* (Sheldrick, 2008 ▶); software used to prepare material for publication: *SHELXTL/PC*.

## Supplementary Material

Crystal structure: contains datablocks I, global. DOI: 10.1107/S1600536810053365/fb2230sup1.cif

Structure factors: contains datablocks I. DOI: 10.1107/S1600536810053365/fb2230Isup2.hkl

Additional supplementary materials:  crystallographic information; 3D view; checkCIF report

## Figures and Tables

**Table 1:** Hydrogen-bond geometry (Å, °) *Cg*1, *Cg*2 and *Cg*3 are the centroids of the N1,N2,C15–C17, C5–C7,C12–C14 and C7–C12 rings, respectively.

*D*—H⋯*A*	*D*—H	H⋯*A*	*D*⋯*A*	*D*—H⋯*A*
C8—H8*A*⋯N1	0.93	2.47	3.047 (3)	120
C8—H8*A*⋯N2	0.93	2.54	3.391 (3)	152
C8—H8*A*⋯*Cg*1	0.93	2.29	2.979 (3)	142
C15—H15*A*⋯*Cg*2^i^	0.99	2.90 (3)	3.731 (3)	142
C4—H4*A*⋯*Cg*3^i^	0.976	2.95	3.824 (3)	150

Symmetry code: (i) 
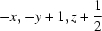
.

**Fig. 1.**
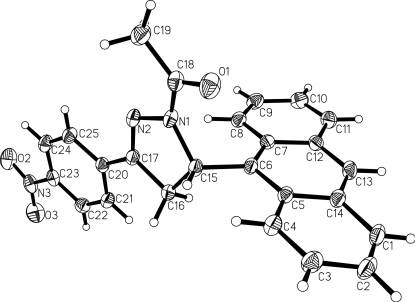
The title molecule, showing the atom-numbering scheme. The displacement ellipsoids are drawn at the 30% probability level.

**Fig. 2.**
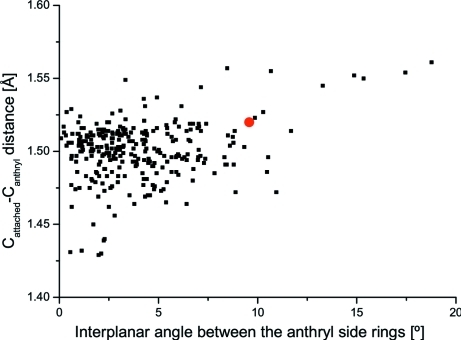
The correlation between the interplanar angles of the side rings in the anthryl fragments retrieved from the Cambridge Crystallographic Database (Allen, 2002; version CSD 5.31 with the last upgrade from Sept. 1 2010) and the length of the bond by which the anthryl is attached to the rest of the molecule. (Cattached and Canthryl correspond to C15 and C6 atoms of the title molecule, respectively.) The title compound is indicated by a red large circle while the rest of the compounds by small black squares.

## supplementary crystallographic information

### Comment

Pyrazoline derivatives are widely studied compounds. Some of them are capable of prototropic tautomerism (Akama *et al.*, 1996). Others show elevated fluorescence. Therefore they have been widely used as fluorescence probes in some elaborated chemosensors (Fahrni *et al.*, 2003) as well as hole-transport materials in electrophotography and electroluminescence (Wei *et al.*, 2007). Here we report the structure of the title compound, a new derivative of pyrazoline.

In the pyrazoline ring, all the atoms are coplanar with the maximum deviation of 0.0258 (14)° for atom N1. The bond length of N2═C17 [1.2865 (31) Å] agrees with normal C═N bond (1.28 Å). The bond distance of N1—N2 [1.3796 (25) Å] conforms to the expected value (Krishna *et al.*, 1999), too. The mean plane of pyrazoline ring makes interplanar angles of 8.80 (15)° and 88.36 (5)° with 4-nitrophenyl ring and the anthryl fragment, respectively.

The most interesting feature of the title structure is the distortion of the anthryl from planarity. The side rings of the anthryl fragment, *i. e.* the benzene rings C1\C2\C3\C4\C5\C14 and C7\C8\C9\C10\C11\C12, contain the angle 9.57 (8)°. In the anthryl fragment, the maximum deviation is 0.126 (3) Å from C4 atom to the mean plane of the ring. It can be related to the repulsion between the methine H15A and the anthryl H4A atoms (Fig. 1). The non-bonding distance between these two hydrogens equals to 1.95 Å. The attached atom H4A is also situated out of the plane of the ring.

Fig. 2 was obtained from the search on the Cambridge Crystallographic Structure Database (Allen, 2002; version CSD 5.31 with the last upgrade from Sept. 1 2010) carried out on the structures with R-factor < 0.05, with no disorder, no errors, with exclusion of the powder diffraction determinations and the polymeric structures or structures containing ions. This plot shows the correlation of the interplanar angle of the side anthryl rings with the C—C distance corresponding to C15—C6 (1.520 Å) in the title structure. It can be seen that the loss of planarity of the anthryl fragment is correlated to the lengthening of the bond by which is the anthryl attached to the the rest of the molecule. The present structure is situated at the extreme of the plot in Fig. 2.

There are present only weak intermolecular interactions in the structure: C—H···N, C—H···π- electron ring interactions (Tab. 1). Moreover, there are also π-electron ring -π-electron ring interactions present in the structure: Between the pyrazoline ring N1\N2\C17\C16\C15 and the benzene C1\C2\C3\C4\C5\C14 ring [symmetry code: -*x*,1-*y*,1/2+*z*] with the centroid-centroid distance equal to 3.7659 (17) Å; between the pyrazoline ring N1\N2\C17\C16\C15 and the benzene C5\C6\C7\C12\C13\C14 ring [symmetry code: *x*, *y*, *z*] with the centroid-centroid distance equal to 3.9477 (15) Å and between the pyrazoline ring N1\N2\C17\C16\C15 and the benzene C5\C6\C7\C12\C13\C14 ring [symmetry code: *-x*, 1-*y*, 1/2+*z*] with the centroid-centroid distance equal to 3.8972 (15) Å.

### Experimental

3-(9-anthryl)-1-(4-nitrophenylprop)-2-en-1-one (3 mmol) and 0.6 g of hydrazine hydrate aqueous solution (1:1 *w*/*w*) were dissolved in 10 ml of glacial acetic acid. The mixture was stirred for 8 h at 391 K to give an orange solid. The product was then isolated and recrystallized from acetonitrile. The average size of light yellow single-crystals of the title structure equalled to 2.0 mm×1.0 mm×1.0 mm.

### Refinement

All the H atoms were discernible in the difference electron density map. Nevertheless all the H atoms except the atoms H4A and H15A the coordinates of which have been freely refined were fully constrained. (The atoms H4A and H15A are involved in the repulsion that plausibly results in the deformation of the anthryl fragment - see the comment section.) The values of the used constraints were following: C_aryl_—H_aryl_ = 0.93, C_methyl_—H_methyl_ = 0.96, C_methylene_—H_methylene_ = 0.97,  C_methine_—H_methine_ = 0.98 Å; *U*_iso_H_aryl/methylene/methine_ = 1.2*U*_eq_C_aryl/methylene/methine_; *U*_iso_H_methyl_ = 1.5*U*_eq_C_methyl_. As there have been present no significant atomic scatterers in the experiment, 2058 Friedel pairs were merged.

### Crystal data

**Table:**

C_25_H_19_N_3_O_3_	*F*(000) = 856
*M**_r_* = 409.43	*D*_x_ = 1.409 Mg m^−^^3^
Orthorhombic, *P**c**a*2_1_	Mo *K*α radiation, λ = 0.71073 Å
Hall symbol: P 2c -2ac	Cell parameters from 3824 reflections
*a* = 22.888 (5) Å	θ = 2.6–25.0°
*b* = 9.4031 (19) Å	µ = 0.09 mm^−^^1^
*c* = 8.9688 (18) Å	*T* = 293 K
*V* = 1930.3 (7) Å^3^	Prism, yellow
*Z* = 4	0.30 × 0.20 × 0.20 mm

### Data collection

**Table:**

Rigaku SCXmini diffractometer	2352 independent reflections
Radiation source: fine-focus sealed tube	2131 reflections with *I* > 2σ(*I*)
graphite	*R*_int_ = 0.062
φ and ω scans	θ_max_ = 27.5°, θ_min_ = 3.3°
Absorption correction: multi-scan (*CrystalClear*; Rigaku, 2005)	*h* = −29→29
*T*_min_ = 0.962, *T*_max_ = 0.982	*k* = −12→12
19272 measured reflections	*l* = −11→11

### Refinement

**Table:**

Refinement on *F*^2^	Secondary atom site location: difference Fourier map
Least-squares matrix: full	Hydrogen site location: difference Fourier map
*R*[*F*^2^ > 2σ(*F*^2^)] = 0.039	H atoms treated by a mixture of independent and constrained refinement
*wR*(*F*^2^) = 0.097	*w* = 1/[σ^2^(*F*_o_^2^) + (0.0488*P*)^2^ + 0.2241*P*] where *P* = (*F*_o_^2^ + 2*F*_c_^2^)/3
*S* = 1.07	(Δ/σ)_max_ < 0.001
2352 reflections	Δρ_max_ = 0.17 e Å^−^^3^
288 parameters	Δρ_min_ = −0.18 e Å^−^^3^
69 restraints	Extinction correction: *SHELXL97* (Sheldrick, 2008), Fc^*^=kFc[1+0.001xFc^2^λ^3^/sin(2θ)]^-1/4^
Primary atom site location: structure-invariant direct methods	Extinction coefficient: 0.018 (2)

### Special details

**Table:**

Geometry. All e.s.d.'s (except the e.s.d. in the dihedral angle between two l.s. planes) are estimated using the full covariance matrix. The cell e.s.d.'s are taken into account individually in the estimation of e.s.d.'s in distances, angles and torsion angles; correlations between e.s.d.'s in cell parameters are only used when they are defined by crystal symmetry. An approximate (isotropic) treatment of cell e.s.d.'s is used for estimating e.s.d.'s involving l.s. planes.
Refinement. Refinement of *F*^2^ against ALL reflections. The weighted *R*- factor *wR* and goodness of fit *S* are based on *F*^2^, conventional *R*-factors *R* are based on *F*, with *F* set to zero for negative *F*^2^. The threshold expression of *F*^2^ > σ(*F*^2^) is used only for calculating *R*-factors(gt) *etc*. and is not relevant to the choice of reflections for refinement. *R*-factors based on *F*^2^ are statistically about twice as large as those based on *F*, and *R*- factors based on ALL data will be even larger.

### Fractional atomic coordinates and isotropic or equivalent isotropic displacement parameters (Å^2^)

**Table:**

	*x*	*y*	*z*	*U*_iso_*/*U*_eq_	
N1	0.09343 (7)	0.5404 (2)	0.2678 (2)	0.0364 (4)	
C5	−0.06548 (9)	0.5860 (2)	0.2243 (2)	0.0319 (5)	
C6	−0.00794 (9)	0.6399 (2)	0.2175 (2)	0.0316 (4)	
N2	0.14083 (8)	0.6030 (2)	0.3366 (2)	0.0360 (4)	
C13	−0.09433 (10)	0.7481 (2)	0.0248 (3)	0.0410 (5)	
H13A	−0.1228	0.7832	−0.0394	0.049*	
C8	0.06219 (10)	0.8147 (2)	0.1055 (3)	0.0390 (5)	
H8A	0.0915	0.7868	0.1711	0.047*	
C17	0.12204 (9)	0.6934 (2)	0.4330 (3)	0.0335 (5)	
C15	0.03652 (9)	0.5881 (2)	0.3311 (3)	0.0337 (5)	
H15A	0.0208 (10)	0.506 (3)	0.387 (3)	0.040*	
C7	0.00641 (9)	0.7476 (2)	0.1140 (2)	0.0326 (5)	
C25	0.22213 (9)	0.7571 (3)	0.5197 (3)	0.0416 (5)	
H25A	0.2374	0.6845	0.4611	0.050*	
C12	−0.03748 (10)	0.7977 (2)	0.0123 (3)	0.0355 (5)	
C23	0.23569 (10)	0.9475 (2)	0.6891 (3)	0.0405 (5)	
C20	0.16176 (9)	0.7811 (2)	0.5216 (3)	0.0344 (5)	
C1	−0.16961 (10)	0.6046 (3)	0.1484 (3)	0.0454 (6)	
H1A	−0.1985	0.6480	0.0915	0.054*	
O3	0.25365 (12)	1.1255 (2)	0.8605 (3)	0.0729 (7)	
C14	−0.10970 (9)	0.6478 (2)	0.1304 (3)	0.0368 (5)	
O1	0.05499 (8)	0.3576 (2)	0.1437 (3)	0.0563 (5)	
C22	0.17679 (11)	0.9742 (3)	0.6936 (3)	0.0453 (6)	
H22A	0.1620	1.0473	0.7523	0.054*	
C4	−0.08379 (10)	0.4732 (3)	0.3199 (3)	0.0400 (5)	
H4A	−0.0558 (12)	0.417 (3)	0.377 (3)	0.048*	
C9	0.07371 (11)	0.9182 (3)	0.0041 (3)	0.0467 (6)	
H9A	0.1101	0.9621	0.0041	0.056*	
C21	0.13977 (10)	0.8900 (3)	0.6090 (3)	0.0421 (6)	
H21A	0.0997	0.9068	0.6110	0.051*	
C16	0.05649 (9)	0.7025 (3)	0.4436 (3)	0.0371 (5)	
H16A	0.0425	0.7960	0.4152	0.045*	
H16B	0.0430	0.6808	0.5435	0.045*	
C3	−0.14067 (10)	0.4332 (3)	0.3298 (3)	0.0463 (6)	
H3A	−0.1510	0.3589	0.3929	0.056*	
C11	−0.02261 (12)	0.9016 (3)	−0.0966 (3)	0.0474 (6)	
H11A	−0.0506	0.9297	−0.1659	0.057*	
N3	0.27495 (10)	1.0378 (2)	0.7773 (3)	0.0515 (6)	
C24	0.25901 (10)	0.8402 (3)	0.6037 (3)	0.0449 (6)	
H24A	0.2991	0.8241	0.6027	0.054*	
O2	0.32753 (9)	1.0208 (2)	0.7630 (3)	0.0698 (7)	
C18	0.09882 (10)	0.4194 (2)	0.1846 (3)	0.0397 (5)	
C2	−0.18442 (10)	0.5023 (3)	0.2462 (4)	0.0497 (6)	
H29A	−0.2234	0.4772	0.2587	0.060*	
C19	0.15940 (11)	0.3706 (3)	0.1485 (4)	0.0528 (7)	
H19A	0.1576	0.2937	0.0783	0.079*	
H19B	0.1784	0.3389	0.2380	0.079*	
H19C	0.1811	0.4480	0.1061	0.079*	
C10	0.03117 (11)	0.9604 (3)	−0.1016 (3)	0.0515 (7)	
H10A	0.0402	1.0280	−0.1737	0.062*	

### Atomic displacement parameters (Å^2^)

**Table:**

	*U*^11^	*U*^22^	*U*^33^	*U*^12^	*U*^13^	*U*^23^
N1	0.0253 (8)	0.0385 (10)	0.0455 (11)	0.0019 (7)	−0.0063 (8)	0.0004 (9)
C5	0.0279 (10)	0.0329 (10)	0.0349 (12)	0.0039 (8)	−0.0028 (9)	−0.0064 (9)
C6	0.0271 (9)	0.0328 (10)	0.0348 (11)	0.0048 (8)	−0.0054 (9)	−0.0017 (9)
N2	0.0266 (9)	0.0383 (10)	0.0430 (10)	−0.0011 (7)	−0.0073 (8)	0.0041 (9)
C13	0.0389 (12)	0.0428 (12)	0.0412 (13)	0.0136 (10)	−0.0119 (11)	−0.0007 (11)
C8	0.0350 (11)	0.0412 (11)	0.0408 (13)	0.0055 (9)	0.0004 (10)	0.0055 (10)
C17	0.0275 (10)	0.0370 (11)	0.0361 (12)	0.0006 (8)	−0.0023 (9)	0.0063 (10)
C15	0.0259 (10)	0.0389 (11)	0.0362 (11)	−0.0015 (9)	−0.0042 (9)	0.0071 (10)
C7	0.0335 (11)	0.0330 (10)	0.0312 (11)	0.0063 (8)	−0.0004 (9)	−0.0024 (9)
C25	0.0313 (11)	0.0448 (13)	0.0486 (14)	0.0004 (9)	−0.0041 (11)	−0.0027 (12)
C12	0.0376 (11)	0.0352 (10)	0.0336 (11)	0.0087 (9)	−0.0035 (10)	−0.0003 (10)
C23	0.0437 (13)	0.0394 (11)	0.0382 (13)	−0.0085 (10)	−0.0099 (11)	0.0081 (10)
C20	0.0305 (10)	0.0387 (11)	0.0340 (11)	−0.0038 (8)	−0.0028 (9)	0.0065 (10)
C1	0.0283 (11)	0.0526 (14)	0.0553 (15)	0.0067 (10)	−0.0114 (11)	−0.0095 (13)
O3	0.0843 (15)	0.0646 (12)	0.0698 (16)	−0.0126 (13)	−0.0163 (12)	−0.0162 (13)
C14	0.0304 (10)	0.0378 (11)	0.0421 (12)	0.0058 (9)	−0.0055 (10)	−0.0091 (10)
O1	0.0478 (10)	0.0497 (10)	0.0713 (13)	−0.0029 (8)	−0.0149 (10)	−0.0094 (10)
C22	0.0483 (14)	0.0478 (13)	0.0399 (13)	0.0014 (11)	−0.0015 (11)	−0.0043 (11)
C4	0.0316 (12)	0.0417 (13)	0.0468 (14)	−0.0006 (9)	−0.0033 (10)	0.0026 (11)
C9	0.0414 (13)	0.0461 (13)	0.0527 (15)	0.0033 (10)	0.0076 (12)	0.0102 (12)
C21	0.0316 (11)	0.0502 (13)	0.0445 (14)	0.0019 (10)	−0.0047 (10)	−0.0013 (11)
C16	0.0287 (10)	0.0493 (13)	0.0333 (12)	0.0008 (9)	−0.0025 (9)	0.0014 (10)
C3	0.0358 (12)	0.0499 (14)	0.0531 (15)	−0.0074 (10)	0.0019 (11)	−0.0012 (12)
C11	0.0510 (14)	0.0498 (13)	0.0413 (13)	0.0145 (11)	−0.0051 (12)	0.0072 (11)
N3	0.0599 (14)	0.0461 (12)	0.0487 (13)	−0.0135 (10)	−0.0163 (12)	0.0074 (11)
C24	0.0304 (11)	0.0498 (13)	0.0546 (16)	−0.0033 (10)	−0.0073 (11)	0.0050 (12)
O2	0.0491 (11)	0.0722 (13)	0.0881 (17)	−0.0182 (10)	−0.0255 (12)	−0.0016 (13)
C18	0.0392 (12)	0.0353 (11)	0.0446 (13)	0.0029 (9)	−0.0087 (10)	0.0026 (11)
C2	0.0269 (10)	0.0579 (15)	0.0642 (17)	−0.0043 (10)	−0.0019 (12)	−0.0107 (14)
C19	0.0482 (14)	0.0493 (14)	0.0609 (17)	0.0122 (11)	−0.0046 (13)	−0.0073 (14)
C10	0.0572 (16)	0.0498 (14)	0.0476 (15)	0.0119 (12)	0.0084 (13)	0.0191 (12)

### Geometric parameters (Å, °)

**Table:**

N1—C18	1.367 (3)	C20—C21	1.384 (3)
N1—N2	1.380 (3)	C1—C2	1.345 (4)
N1—C15	1.490 (3)	C1—C14	1.439 (3)
C5—C6	1.412 (3)	C1—H1A	0.9300
C5—C4	1.427 (3)	O3—N3	1.214 (3)
C5—C14	1.440 (3)	O1—C18	1.216 (3)
C6—C7	1.413 (3)	C22—C21	1.386 (3)
C6—C15	1.520 (3)	C22—H22A	0.9300
N2—C17	1.287 (3)	C4—C3	1.358 (3)
C13—C14	1.382 (4)	C4—H4A	0.97 (3)
C13—C12	1.387 (3)	C9—C10	1.416 (4)
C13—H13A	0.9300	C9—H9A	0.9300
C8—C9	1.358 (3)	C21—H21A	0.9300
C8—C7	1.426 (3)	C16—H16A	0.9700
C8—H8A	0.9300	C16—H16B	0.9700
C17—C20	1.462 (3)	C3—C2	1.409 (4)
C17—C16	1.506 (3)	C3—H3A	0.9300
C15—C16	1.544 (3)	C11—C10	1.350 (4)
C15—H15A	0.99 (3)	C11—H11A	0.9300
C7—C12	1.436 (3)	N3—O2	1.221 (3)
C25—C24	1.375 (3)	C24—H24A	0.9300
C25—C20	1.400 (3)	C18—C19	1.496 (3)
C25—H25A	0.9300	C2—H29A	0.9300
C12—C11	1.423 (4)	C19—H19A	0.9600
C23—C22	1.372 (3)	C19—H19B	0.9600
C23—C24	1.374 (4)	C19—H19C	0.9600
C23—N3	1.468 (3)	C10—H10A	0.9300
			
C18—N1—N2	121.89 (18)	C23—C22—C21	118.7 (2)
C18—N1—C15	122.51 (18)	C23—C22—H22A	120.6
N2—N1—C15	112.89 (18)	C21—C22—H22A	120.6
C6—C5—C4	124.50 (19)	C3—C4—C5	121.8 (2)
C6—C5—C14	119.1 (2)	C3—C4—H4A	116.5 (16)
C4—C5—C14	116.45 (19)	C5—C4—H4A	121.6 (16)
C5—C6—C7	120.14 (18)	C8—C9—C10	121.0 (2)
C5—C6—C15	118.71 (19)	C8—C9—H9A	119.5
C7—C6—C15	120.98 (18)	C10—C9—H9A	119.5
C17—N2—N1	108.61 (17)	C20—C21—C22	120.7 (2)
C14—C13—C12	121.6 (2)	C20—C21—H21A	119.7
C14—C13—H13A	119.2	C22—C21—H21A	119.7
C12—C13—H13A	119.2	C17—C16—C15	102.38 (18)
C9—C8—C7	121.8 (2)	C17—C16—H16A	111.3
C9—C8—H8A	119.1	C15—C16—H16A	111.3
C7—C8—H8A	119.1	C17—C16—H16B	111.3
N2—C17—C20	122.01 (19)	C15—C16—H16B	111.3
N2—C17—C16	114.39 (19)	H16A—C16—H16B	109.2
C20—C17—C16	123.6 (2)	C4—C3—C2	121.3 (3)
N1—C15—C6	115.24 (19)	C4—C3—H3A	119.4
N1—C15—C16	101.51 (16)	C2—C3—H3A	119.4
C6—C15—C16	114.36 (18)	C10—C11—C12	121.4 (2)
N1—C15—H15A	106.1 (14)	C10—C11—H11A	119.3
C6—C15—H15A	110.3 (15)	C12—C11—H11A	119.3
C16—C15—H15A	108.6 (16)	O3—N3—O2	123.3 (2)
C6—C7—C8	124.09 (19)	O3—N3—C23	118.6 (2)
C6—C7—C12	119.35 (19)	O2—N3—C23	118.1 (2)
C8—C7—C12	116.5 (2)	C23—C24—C25	118.9 (2)
C24—C25—C20	120.5 (2)	C23—C24—H24A	120.5
C24—C25—H25A	119.7	C25—C24—H24A	120.5
C20—C25—H25A	119.7	O1—C18—N1	119.2 (2)
C13—C12—C11	120.7 (2)	O1—C18—C19	123.6 (2)
C13—C12—C7	119.6 (2)	N1—C18—C19	117.2 (2)
C11—C12—C7	119.6 (2)	C1—C2—C3	119.8 (2)
C22—C23—C24	122.2 (2)	C1—C2—H29A	120.1
C22—C23—N3	118.7 (2)	C3—C2—H29A	120.1
C24—C23—N3	119.2 (2)	C18—C19—H19A	109.5
C21—C20—C25	119.0 (2)	C18—C19—H19B	109.5
C21—C20—C17	119.93 (19)	H19A—C19—H19B	109.5
C25—C20—C17	121.1 (2)	C18—C19—H19C	109.5
C2—C1—C14	121.0 (2)	H19A—C19—H19C	109.5
C2—C1—H1A	119.5	H19B—C19—H19C	109.5
C14—C1—H1A	119.5	C11—C10—C9	119.4 (2)
C13—C14—C1	120.8 (2)	C11—C10—H10A	120.3
C13—C14—C5	119.8 (2)	C9—C10—H10A	120.3
C1—C14—C5	119.3 (2)		

### Hydrogen-bond geometry (Å, °)

**Table:**

Cg1, Cg2 and Cg3 are the centroids of the N1,N2,C15–C17, C5–C7,C12–C14 and C7–C12 rings, respectively.

**Table:**

*D*—H···*A*	*D*—H	H···*A*	*D*···*A*	*D*—H···*A*
C8—H8A···N1	0.93	2.47	3.047 (3)	120.
C8—H8A···N2	0.93	2.54	3.391 (3)	152.
C8—H8A···Cg1	0.93	2.29	2.979 (3)	142
C15—H15A···Cg2^i^	0.99	2.90 (3)	3.731 (3)	142
C4—H4A···Cg3^i^	0.976	2.95	3.824 (3)	150

Symmetry codes: (i) −*x*, −*y*+1, *z*+1/2.
